# The Management of Open Apex Using a Bioactive Material: A Case Report

**DOI:** 10.7759/cureus.61296

**Published:** 2024-05-29

**Authors:** Mrinal Nadgouda, Aditya Patel, Manoj Chandak, Shweta Sedani, Swayangprabha Sarangi

**Affiliations:** 1 Conservative Dentistry and Endodontics, Sharad Pawar Dental College and Hospital, Datta Meghe Institute of Higher Education and Research, Wardha, IND

**Keywords:** ceramic crown, backfill, biodentine material, bioactive material, open apex

## Abstract

The management of teeth with open apices poses unique challenges in endodontics, requiring effective strategies to promote continued root development and maintain pulp vitality. This abstract explores the utilization of bioactive materials in the treatment of open apices, specifically focusing on their role in achieving optimal outcomes. Bioactive materials, such as Biodentine (Septodont, Saint-Maur-des-Fossés, France), have gained prominence for their favourable physiochemical properties, biocompatibility, and ability to stimulate dentinogenesis. The application of a bioactive material as an apical plug not only addresses immediate concerns but also contributes to long-term health and stability. This abstract reviews relevant literature, discusses clinical cases, and emphasizes the importance of tailoring treatment plans to the individual characteristics of open apex cases. The findings underscore the promising role of bioactive materials in reshaping the landscape of endodontic interventions for teeth with open apices, highlighting their potential to enhance both clinical and radiographic success.

## Introduction

The concept of an open apex in endodontics refers to the incomplete development of the root apex, presenting a distinctive set of challenges in the diagnosis and treatment of dental pathology [1]. Teeth with open apices are commonly encountered in clinical practice, particularly in cases involving trauma, developmental anomalies, or immature permanent teeth [2]. The unique anatomical characteristics of open apex teeth, marked by a lack of closure at the root tip, pose difficulties in conventional endodontic procedures, necessitating innovative and evolving treatment strategies. The open apex condition arises when the normal process of root maturation is disrupted, hindering the formation of a fully developed apical foramen [3]. This phenomenon can be attributed to factors such as trauma during tooth development, genetic predispositions, or certain systemic conditions affecting dental formation. Gaining insight into the fundamental causes of open apices is essential for customizing successful treatment strategies that not only tackle immediate issues but also ensure the long-term health and stability of the implicated tooth [3]. In this context, advancements in endodontic techniques, regenerative therapies, and materials have significantly expanded the armamentarium available to clinicians dealing with open apex cases. Conventional methods, like apexification, have been augmented by regenerative endodontic interventions designed to encourage ongoing root development and maintain pulp vitality [4]. This introduction lays the groundwork for a more in-depth investigation into the intricate aspects of open apex cases. By scrutinizing the distinct challenges they present and the inventive solutions that have surfaced in the realm of endodontics, we acquire valuable insights into the dynamic landscape of dental care and the quest for optimal outcomes in patients with open apex conditions.

## Case presentation

**Figure 1:**
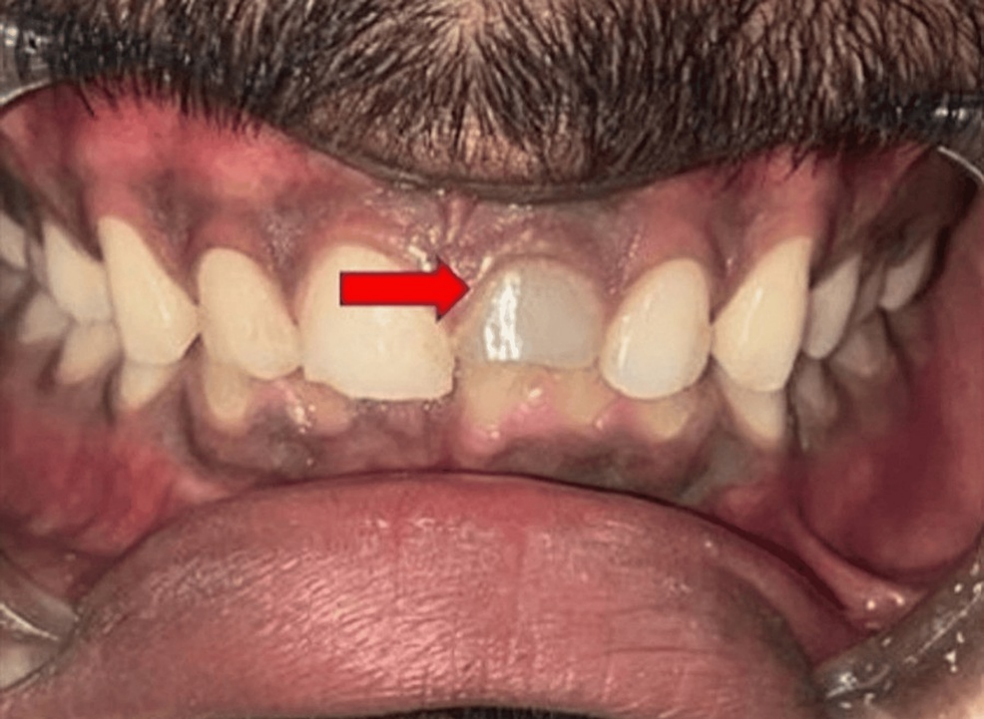
Preoperative clinical photograph

A 21-year-old male patient presented to the Department of Conservative Dentistry and Endodontics with the chief complaint of a broken upper front tooth and unaesthetic discolouration for nine years. Clinical investigation revealed pulpal exposure, which caused no tenderness on the percussion when checked in both horizontal and vertical directions. There was no history of swelling, night pain, fever, mobility, or a draining sinus (Figure 1).

**Figure 2:**
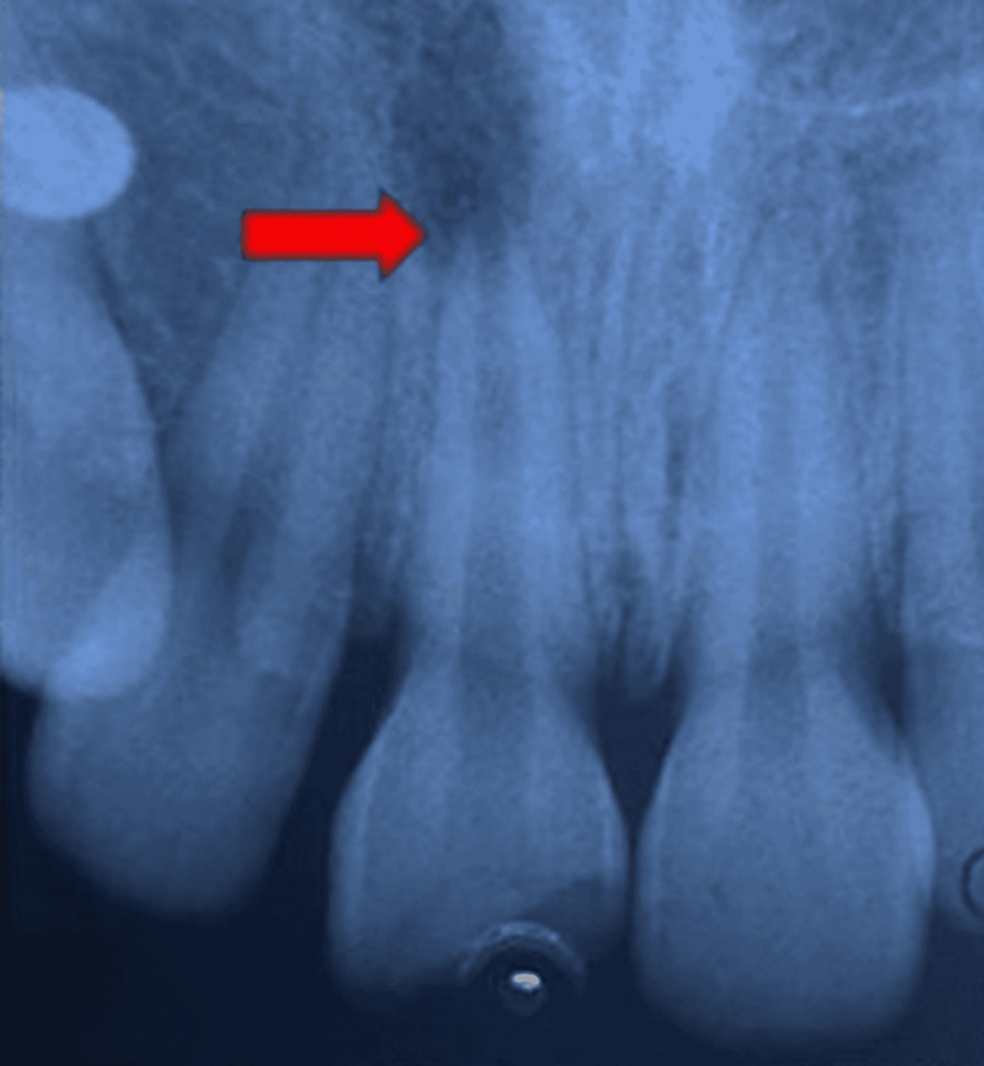
Preoperative radiograph where the arrow is indicating incomplete root formation along with a periapical lesion.

Radiographic examination showed an incomplete root formation along with a periapical lesion located lateral to the apex of tooth 21 (Figure 2).

**Figure 3:**
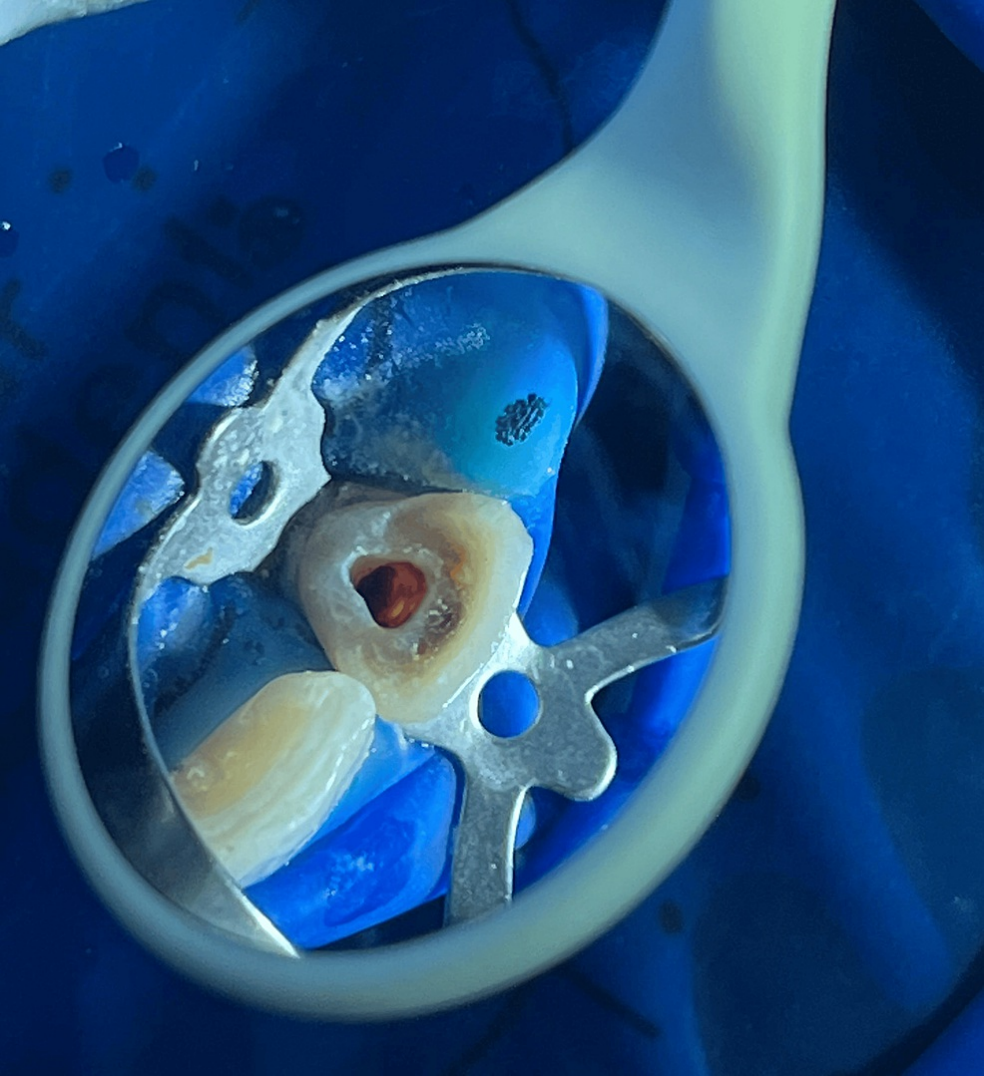
Access opening was performed utilizing a round bur.

Utilizing electric pulp testing (Digitest II; Parkell Inc., Edgewood, USA) yielded no response, indicating total pulp necrosis. The patient was apprised of the proposed treatment approach, and informed consent was duly obtained for undergoing a root canal procedure. The chosen course of action involved implementing an apical plug for closure, as the treatment plan since the apex was incompletely formed. In the initial appointment, tooth 21 was isolated using a rubber dam. The access opening was performed utilizing a round bur (BR-45; Mani, Tokyo, Japan) (Figure 3).

**Figure 4:**
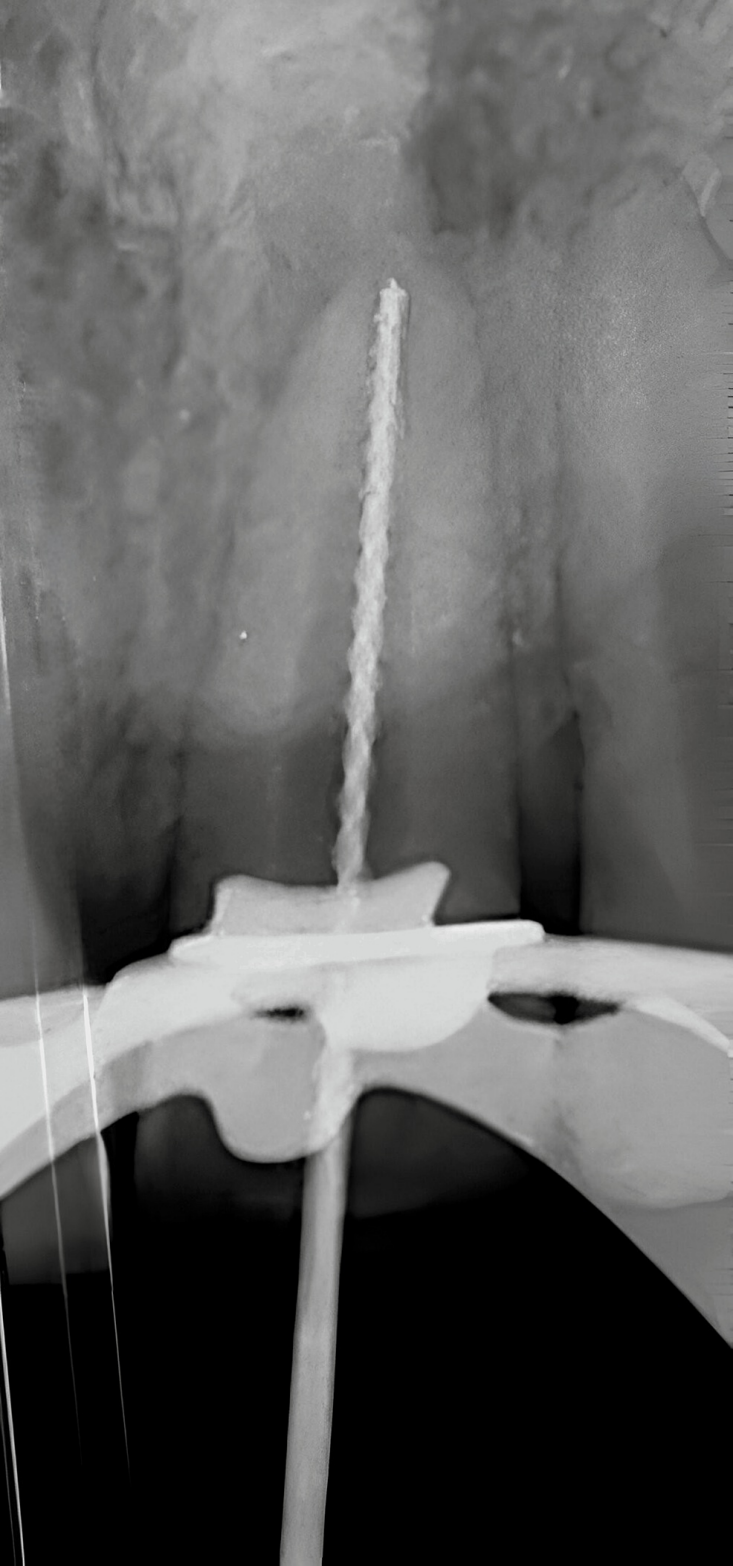
Working length determination

Working length was measured using a hand 15K file (Mani, Tokyo, Japan) and was electronically verified using an apex locator (J. Morita, Kyoto, Japan) and confirmed radiographically which was found to be 23 mm (Figure 4).

**Figure 5:**
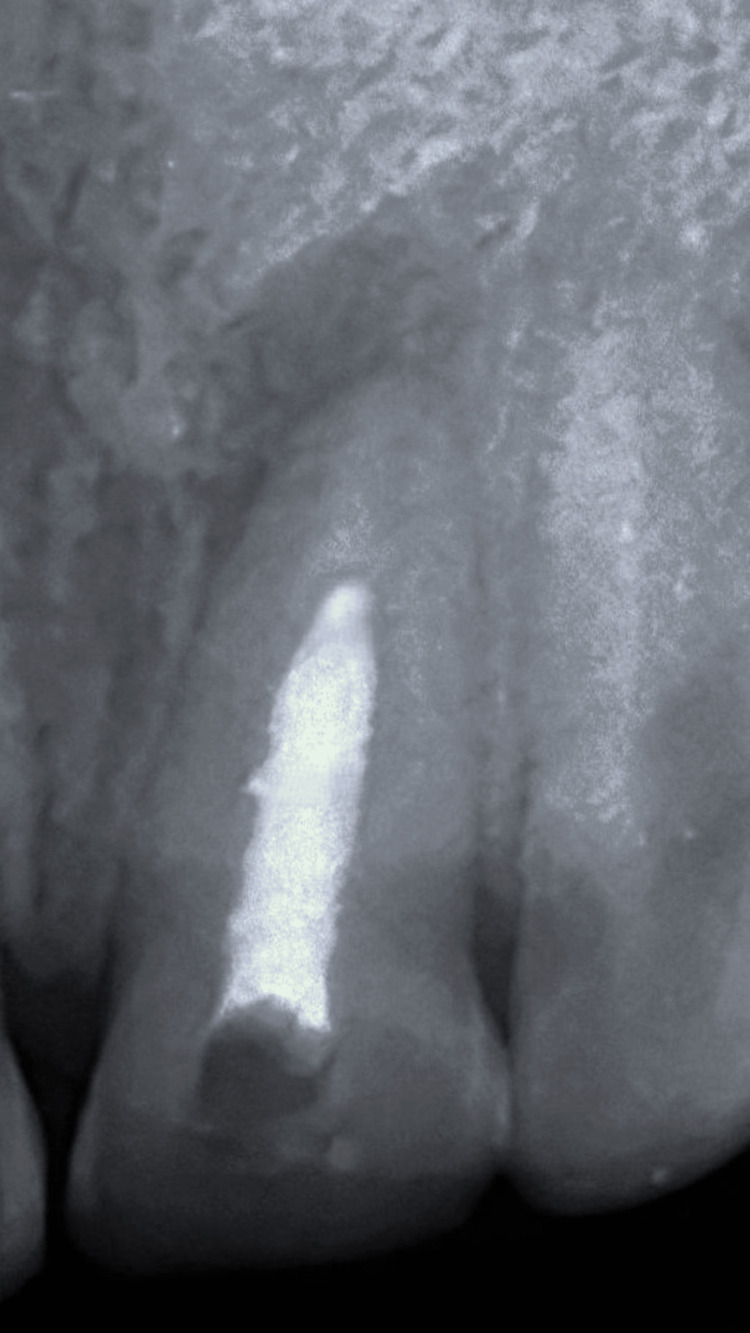
Sealing of apex using Biodentine and obturation done using thermoplasticized gutta-percha.

Biomechanical preparation was done using the crown-down method up to an 80K file (Mani, Tokyo, Japan). Following this, a calcium hydroxide (CaOH_2_) dressing was given to tooth 21, and the patient was recalled after seven days. During the second visit, the patient was completely asymptomatic, and it was decided to proceed with further treatment. Biodentine (Septodont, Saint-Maur-des-Fossés, France) was used for the apical plug with tooth 21. With the help of a plugger, Biodentine was inserted into the canal up to 5 mm from the working length. It was then decided to obturate the canal on the same visit using the thermoplasticized technique (System B; Sybron Endo, Orange, USA) (Figure 5).

**Figure 6:**
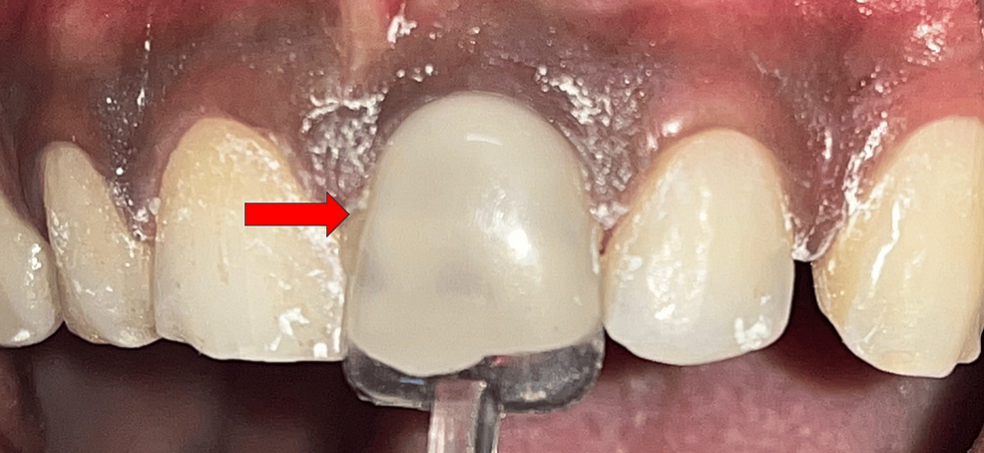
Crown build-up done using packable composite with the help of a template.

The post-endodontic restoration was done with 21 using a micro-hybrid composite (Spectrum; Dentsply Sirona, York, USA) with the help of a template (Figure 6).

**Figure 7:**
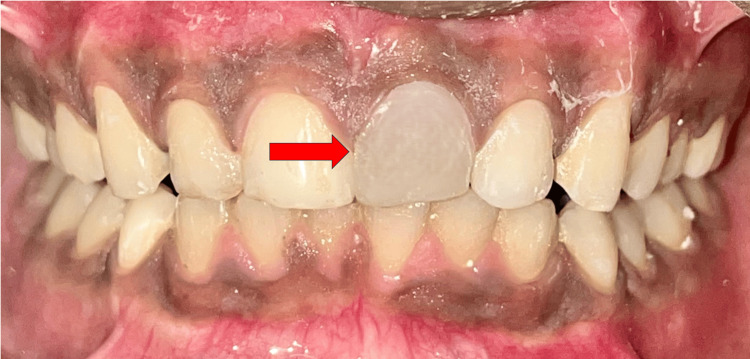
Post-operative clinical photograph after composite build-up.

After the removal of the template, finishing and polishing of the composite buildup were done (Figure 7).

**Figure 8:**
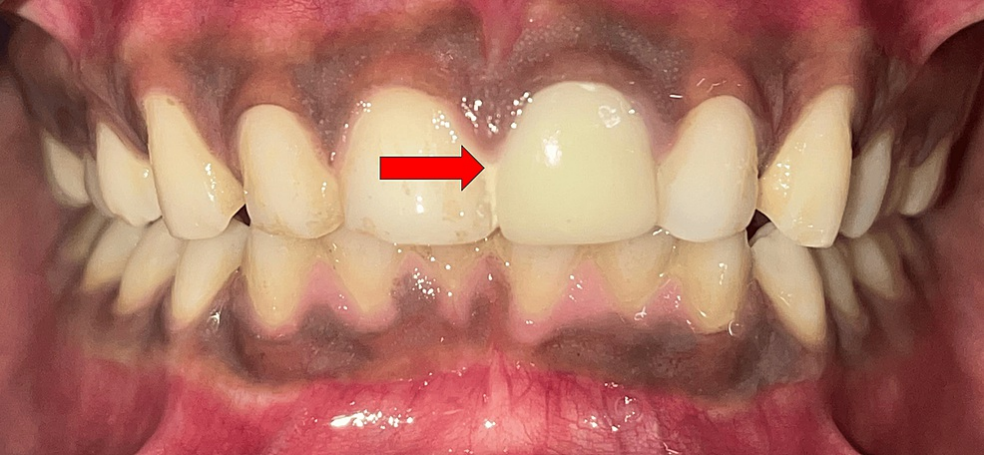
Cementation of all ceramic prosthesis

Lastly, an all-ceramic prosthesis was cemented for 21 (Figure 8).

**Figure 9:**
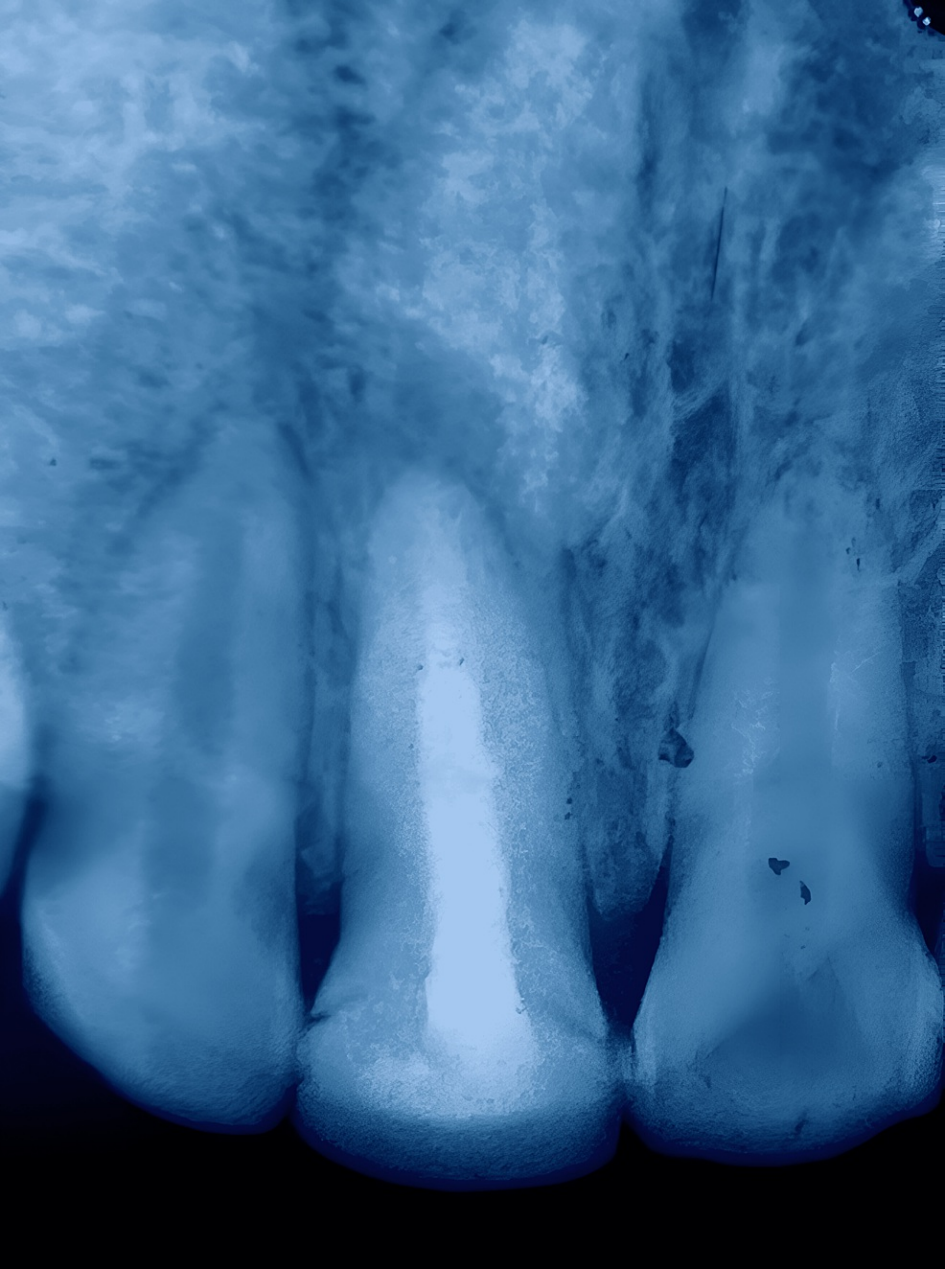
Follow-up after six months where a significant amount of healing was appreciated with no widening of the periodontal ligament.

The patient was recalled for follow-up after six months. The post-operative response of the patient favoured showing no signs of postoperative pain, closure of apex and a subsequent amount of healing. Overall the patient appreciated the visible colour changes which faded significantly after placement of definitive prosthesis with tooth 21. There was no active periapical radiolucency visible on the intraoral periapical radiograph (Figure 9).

## Discussion

The establishment of a calcified barrier through extended calcium-hydroxide treatment was the prevailing method used to attain a biological seal in teeth with incomplete apical formation [5]. A root canal with thin, delicate walls tends to be wide with an open apex [6], which complicates the instrumentation process and inhibits the formation of an adequate apical stop [7]. To address this issue, it is crucial to create an artificial apical barrier or close the apical foramen with calcified tissue. This allows for the condensation of the filling material and ensures proper apical sealing [8]. To mitigate these challenges, numerous studies have advocated for a one-visit apexification approach [9] involving the use of Biodentine, which has emerged as a viable alternative due to its favourable physiochemical properties, biocompatibility [10], and notable clinical success rates [11]. Biodentine, a modern calcium silicate-based material, was developed to maintain the beneficial properties and clinical applications of mineral trioxide aggregate (MTA) without its adverse characteristics. Its enhanced clinical performance allows for safer handling and protection, eliminates the need for a two-step obturation, and features a faster setting time, thereby reducing the risk of bacterial infection. Consequently, Biodentine is considered superior to MTA.

Subsequent to this procedure, the canal can be obturated, and a coronal restoration can be applied [12]. Hence, for the cases described here, an apical plug obturation was the best approach since both needed immediate restoration. In this case report, the justification for employing an intracanal medicament before the ultimate obturation was primarily aimed at reducing the bacterial count, given the presence of diverse bacterial combinations in the root canal system of necrotic teeth [13]. CaOH_2_ possesses the capability to establish an antibacterial environment, thereby aiding in the decontamination of the pulp cavity [14]. Furthermore, due to its heightened pH, the proactive application of CaOH_2_ dressings becomes imperative to establish favourable conditions for the curing of Biodentine and augment its characteristics [15]. The treatment outcome constitutes a crucial aspect of evidence-based practice [16].

In the past, there have been published case reports documenting the occurrence of biological apical closure following root canal filling. A study conducted in vivo on dogs [17] reported the formation of an apical calcified barrier in all teeth treated with a Biodentine plug. Similarly, in a case-control study of 50 patients, apexification with Biodentine showed an 83% success rate, emphasizing the predictability and prognosis of the treatment. However, the success of the treatment is directly related to the diameter of the foramen, sealing ability, and correct adaptation of the used material [18]. Adel et al. emphasized that enlarging the diameter of the apical foramen or reducing the thickness of the apical plug leads to a substantial increase in the apical microleakage of barriers [19].

In a dye leakage study comparing different depths of Biodentine, 4-mm-thick material showed significantly more effectiveness [20], which was applied to the case in this report. Biodentine was applied using hand pluggers for a controlled adaptation of the material. To achieve a good seal and retention during orthograde placement of Biodentine in teeth with open apices, the delivery technique could be improved [21]. However, an apical leakage study comparing various insertion techniques showed similar sealing abilities when Biodentine was placed with pluggers, paper points, or ultrasonic tips [22]. As outlined in the documented instances, the application of Biodentine showcased both clinical and radiographic success during follow-up assessments. Deciding to utilize an apical plug arises as a pragmatic treatment option for overseeing teeth with incomplete root development requiring prompt restoration.

## Conclusions

In conclusion, the management of a tooth with an open apex involves a thoughtful consideration of treatment options, a realistic assessment of prognosis, and a commitment to long-term follow-up. Advances in regenerative endodontic procedures offer promising alternatives, but the choice of treatment should be based on individual case characteristics and clinician expertise. Regular monitoring and continued research will contribute to refining and advancing the field of managing teeth with open apices.
